# Sensitive detection of copy number alterations in low-pass liquid biopsy sequencing data

**DOI:** 10.1093/bib/bbag111

**Published:** 2026-03-16

**Authors:** Lotta Eriksson, Eszter Lakatos

**Affiliations:** Department of Mathematical Sciences, Chalmers University of Technology, Chalmers Tvärgata 3, 412 58 Gothenburg, Sweden; Department of Mathematical Sciences, University of Gothenburg, Chalmers Tvärgata 3, 412 58 Gothenburg, Sweden; Department of Mathematical Sciences, Chalmers University of Technology, Chalmers Tvärgata 3, 412 58 Gothenburg, Sweden; Department of Mathematical Sciences, University of Gothenburg, Chalmers Tvärgata 3, 412 58 Gothenburg, Sweden

**Keywords:** copy number alterations, liquid biopsies, low-pass sequencing, Bayesian changepoint detection

## Abstract

Liquid biopsies, coupled with analysis of copy number alterations (CNAs), have emerged as a promising tool for non-invasive monitoring of cancer progression and tumor composition. However, methods utilizing CNA data from liquid biopsies are limited by the low signal in the samples, caused by a low percentage of cancer DNA in the blood, and inherent noise introduced in the sequencing. To address this challenge, we developed BayesCNA, a method designed to improve signal extraction from low-pass liquid biopsy sequencing data, by utilizing a Bayesian changepoint detection algorithm. We use information of the posterior changepoint probabilities to identify likely changepoints, where a changepoint indicates a shift in the copy number state. The signal is then reconstructed using the identified partition. We show the effectiveness of the method on synthetically generated datasets and compare the method with state-of-the-art bioinformatics tools under noisy conditions. Our results show that this novel approach increases sensitivity in detecting CNAs, particularly in low-quality cases.

## Introduction

Routine sampling and sequencing of patients’ tumors are crucial for enabling precision medicine. However, traditional tissue-based biopsies are invasive and repeatedly collecting representative samples is often impractical, especially for patients with metastatic cancer. Acquiring tissue samples during treatment to monitor tumor response and relapse is particularly challenging. To address the challenges with traditional biopsies, recent oncology research has shifted towards the analysis of bodily fluids for tumor-derived components, referred to as liquid biopsies [1]. Liquid biopsies are promising for longitudinal and minimally invasive monitoring of cancer dynamics, mainly by utilizing cell-free DNA (cfDNA) present in blood samples i.e. released by necrosis or apoptosis of the cell [2]. Tumor-derived cfDNA is correlated with the stage of the disease [3, 4], and offers monitoring and (early) diagnostic potential on par with radiographic evidence or other tissue/blood-based markers [5–9], while being less invasive and producing a comprehensive overview of systemic disease.

Copy number alterations (CNAs) are structural variations that result in discrete gains or losses of large genomic regions. CNAs are prevalent in human cancers and play an important role in cancer progression by activating oncogenes and inactivating tumor suppressors [10–12]. CNAs are not only wide-spread, but—unlike point mutations—are often exclusive to tumor cells, making them a good tool for cancer detection and tracking. Moreover, CNAs can be detected using low-pass whole-genome sequencing (lpWGS), typically performed at coverages of 0.1–5$\times$, making them an affordable biomarker applicable even in scenarios with little or low-quality biological material. Hence, liquid biopsies coupled with the analysis of CNAs are a promising tool for non-invasive monitoring of cancer progression and tumor composition. However, tumor cells are not the only contributor to the DNA pool in the blood, where healthy DNA often comes in a high proportion. Since healthy DNA does not carry the genomic alterations present in cancer cells, the signal in the sample is reduced, making detection of CNAs challenging. Although a wide range of methods are available for CNA detection in tissue biopsies [13–16], including lpWGS samples, these are hampered by the low signal commonly observed in liquid biopsies and tend to break down in low purity scenarios [17]. Methods designed for liquid biopsies [18] offer a wider range of detection, but still can be challenged when low purity is accompanied with low coverage [17]. To address these shortcomings, we developed BayesCNA, a method that utilizes Bayesian changepoint (BCP) detection for sensitive detection of CNAs in low-quality liquid biopsy sequencing data.

Changepoint detection is the problem of identifying times or spatial positions where the probability distribution of a stochastic process or time series changes abruptly. Changepoint detection is used in various fields, including medicine and finance; see, e.g. [19, 20]. In the setting of CNA detection, changepoints correspond to the boundaries of segments, contiguous regions of the genome with identical copy number state. Accurate detection of changepoints is therefore crucial for correct partitioning of the genome and identification of altered copy number regions. Information about the location of CNAs is important in longitudinal study of cancer dynamics, where we are interested in studying how segments change over time, e.g. to estimate the subclonal composition of a tumor at different timepoints [21]. Changepoint detection has been widely used in the analysis of CNAs, and the most popular approaches are circular binary segmentation (CBS) [22] and models based on a Hidden Markov Model (HMM) [18]. However, CBS struggles to detect changepoints when the change in mean is small and when the changed segment is short. HMM-based methods, on the other hand, require a predefined set of possible states, which are often unknown *a priori*. An alternative to traditional changepoint methods is BCP detection, introduced by Barry and Hartigan [23] as a Bayesian approach to the changepoint problem. BCP does not require any assumptions on the number of copy number states and computes the full posterior distributions rather than point estimates, reflecting the uncertainty in the locations of CNAs or copy number states. Furthermore, Erdman and Emerson made a $\mathcal{O}(n)$ MCMC implementation of the model, which allows for fast denoising, even for high-resolution data [19], and showed that BCP works well for segmentation of microarray data.

In this work, we introduce BayesCNA, a segmentation and copy number quantification method designed for low-quality liquid biopsy samples. BayesCNA is designed to take as input either unprocessed read counts or the output of preprocessing performed by state-of-the-art pipelines [16, 18]. It utilizes information about posterior changepoint probabilities estimated using BCP to extract likely positions of CNA segment boundaries from these data. Based on the segmentation gained from BCP, we derive a (floating point) copy number profile of (potentially corrected or centered) read depth ratios that may be used as an input to absolute copy number quantification algorithms. Changepoints in the genome that do not induce a significant change in the segment medians are filtered away to avoid oversegmentation of the genome and focus on high-confidence alterations. We show that BayesCNA provides high-recall segmentation and accurate CNA detection using synthetic profiles and *in silico* sequencing results. Finally, we demonstrate the use of BayesCNA on experimental cell-line and patient data where it achieves high concordance between detected CNAs in a baseline and low-quality replicates.

## Materials and methods

### Bayesian changepoint detection

Let $\mathbf{X} = \{X_{i}\}_{i = 1}^{n}$ denote the data, where $X_{i}$ is the observed copy number values in a genomic bin, and $n$ the number of bins. For example, $\mathbf{X}$ may represent GC- and mappability corrected read depth information. The model assumes that there exists a partition of the genomic bins such that the mean is shared by all bins within each segment, corresponding to the copy number state of that segment. The observations are assumed to be independent $\mathcal{N}(\mu _{i}, \sigma ^{2})$, where $\mu _{i}$ is segment specific but $\sigma ^{2}$ is shared throughout the genome. We assume that the noise in the samples is dominated by normal contamination, which is constant in the sample, making the assumption of equal variance reasonable. The prior of the mean $\mu _{ij}$ of a segment spanning between positions $i + 1$ and $j$ is assumed to be $\mathcal{N}(\mu _{0}, \sigma _{0}^{2} / (j - i))$, where $\mu _{0}$ and $\sigma _{0}^{2}$ are hyperparameters. Note that the variance of the prior depends on the segment length, such that larger deviations from $\mu _{0}$ are expected in short blocks. This assumption is suitable for CNAs since small focal changes often show more copy number states and are also more affected by measurement noise. An exact implementation of Barry and Hartigan’s procedure is possible, but computationally expensive. Therefore, Erdman and Emerson [24] implemented an MCMC approximation and later reduced the computational complexity from $\mathcal{O}(n^{3})$ to $\mathcal{O}(n)$ [19]. For theoretical details and notes on prior specifications, we refer the reader to the original papers.

A partition is denoted as $\rho = (U_{i})_{i = 1}^{n}$ where $U_{i} \in \{0, 1\}$ and $U_{i} = 1$ is the indicator of a changepoint at position $i + 1$. Using MCMC, an approximate sample of $\rho$ is generated in the following manner: initialize $U_{i} = 0$ for all $i < n$ and $U_{n} \equiv 1$. A new partition is generated by iterating through the current partition, and at each position $i$ assign $U_{i} = 1$ with probability $p_{i}$ where


1 \begin{eqnarray*} \frac{p_{i}}{1 - p_{i}} &= \frac{\mathbb{P}(U_{i} = 1 \mid \boldsymbol{X}, U_{j}, j \neq i)}{\mathbb{P}(U_{i} = 0 \mid \boldsymbol{X}, U_{j}, j \neq i)},\end{eqnarray*}

whose exact analytical form was derived by Barry and Hartigan [23] and simplified by Erdman and Emerson as incomplete beta integrals to ensure numerical stability for long sequences [24]. After iterating through the partition, the posterior means are updated conditionally on the current partition. Let $\mathbf{U}$ be a matrix whose rows $U^{(m)}, \, m = 1, \dotsc , M$ consist of MCMC partitions with $U_{i}^{(m)}$ being the indicator of bin $i + 1$ being a changepoint in partition $m$ and $M$ is the number of MCMC iterations after removing the burnin period. The posterior probability $p^\ast _{i}$ that a genomic bin $i + 1$ is a changepoint is estimated as


2 \begin{eqnarray*}& p^\ast_{i} = \frac{1}{M} \sum_{m = 1}^{M} \boldsymbol{1}\{U_{i}^{(m)} = 1\}.\end{eqnarray*}

The method is implemented in the R package bcp by Erdman and Emerson.

### Segmentation and postprocessing

In order to derive copy number profiles for downstream analysis from changepoint detection, segmentation is necessary, i.e. dividing the genome into segments of equal copy number states. We utilize the posterior changepoint probabilities to obtain a probable partition of the genome. Based on $\mathbf{U}$, the most probable partition $\hat{\rho }$ can be estimated by computing the most frequent partition among the samples. However, we found that the frequency of each partition is low, due to the non-sharp boundaries in the data as a consequence of high noise levels, which reduced the segmentation accuracy. To circumvent this issue, we instead utilize information about the posterior changepoint probabilities for extracting likely positions of CNAs in the genome. We filter away noise in the posterior changepoint probabilities by setting


3 \begin{eqnarray*}& p_{i}^\ast = \begin{cases} p_{i}, \quad p_{i}> \varepsilon \\ 0, \quad \textrm{else} \end{cases}, \quad \varepsilon \in [0, 1].\end{eqnarray*}

The parameter $\varepsilon$ can either be fixed for all samples or chosen as, e.g. 10% of the largest posterior changepoint probability, to account for differences between samples. When using such a cutoff, there is a risk of over-segmenting the genome, i.e. introducing too many changepoints and breaking up genomic segments with identical true copy number states. Especially in noisy situations, changepoints are found due to local trends in the data. Therefore, we undo changepoints that are not at least $\eta$ standard deviations apart.

### BayesCNA workflow

**Figure 1:**
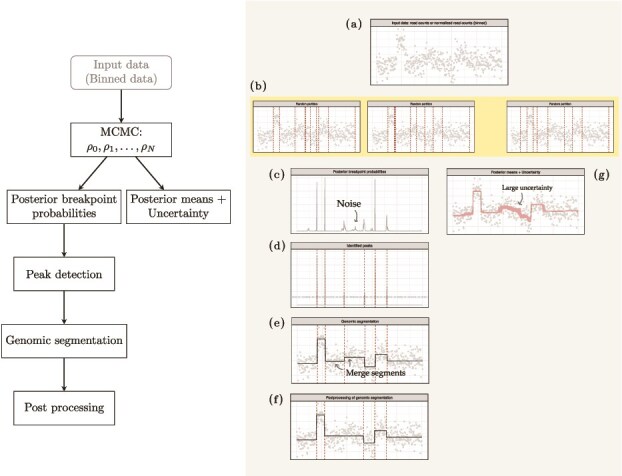
Outline of the estimation method BayesCNA. The left panel shows a flowchart of the individual steps and the right chart illustrates the corresponding steps on simulated input data. (a) BayesCNA takes any genomic binned data (e.g. GC-corrected log2ratios) as input. Here, we use simulated data of chromosome 1 with artificially introduced CNAs. (b) BCP detection is carried out in an MCMC approximation. Examples of three random partitions generated in the MCMC step for the example data. The vertical dashed lines indicate the changepoints in the generated partition. (c) Posterior changepoint probabilities are computed for each genomic bin. Low posterior changepoint probabilities are a consequence of noise in the data. (d) Posterior changepoint probabilities are filtered to be $>\varepsilon$ (horizontal dashed line) and processed with a peak detection algorithm to identify changepoints (dashed vertical lines). (e) Segment-wise copy number values are computed as the conditional means based on the identified partition. (f) Genomic segmentation is further processed to eliminate oversegmentation by merging segments that cause too small change in median. (g) Posterior mean of the copy number profile and 95% CI is also computed directly from the MCMC step, using 2000 MCMC samples.

In Fig. 1, we outline BayesCNA and its required input and output. BayesCNA works for any data formatted as a floating point input vector, and as such can be applied to any binned genomic data, regardless of the preprocessing procedure applied, e.g. raw binned read counts or log2ratios. However, as it assumes that the inputted measurements follow a Gaussian distribution, we recommend preprocessing that eliminates any known biases, such as introduced by GC-content or mappability issues. We use the implementation by Erdman and Emerson [19] to generate a sequence of genome partitions and estimate the posterior changepoint probabilities and means. To gain insight into the uncertainty associated with the estimated mean, we also compute a 95% credible interval (CI) of the mean. Due to the noisy nature of the data, low changepoint probabilities are filtered away before running a peak detection algorithm to detect genomic bins of high posterior changepoint probability. The peak detection yields a set of changepoints, which is then used to reconstruct the copy number profile by computing segment levels as the medians between consecutive changepoints. We prefer the median over the mean as it is more robust to outliers. Changepoints introduced that do not result in a significant shift in median are removed as these are expected to be a consequence of the noise. In summary, BayesCNA identifies segment boundaries and assigns continuous values to each identified segment resulting in a reconstructed quantitative copy number profile. The output of BayesCNA depends on the input data used, but if following standard processing pipelines, it is most often normalized log2-ratios. This resulting signal can then be used in downstream analyses to estimate tumor content or obtain absolute copy numbers [21, 25].

### Simulation of evaluation datasets

We construct a synthetic dataset with artificial copy number profiles with $n = 1000$ genomic bins. For each sample, the segment length is drawn uniformly from $L \in \{30, 31, \dotsc , 50\}$ and each segment is assigned a copy number state $C \in \{1, 2, \dotsc , 5\}$ with probabilities $\{0.09, 0.5, 0.27, 0.09, 0.05\}$ (rounded to two decimals). We control the sample purity $P \in \{0.05, 0.1, \dotsc , 0.5\}$ by averaging the generated signal and a constant value of 2 (corresponding to contamination from healthy DNA), with weights $P$ and $1-P$, respectively. To reflect scenarios of different sequencing depths/coverages, we use four noise levels $\{0.5, 1, 2, 4\}$, as described in [21]. Noise level 1 roughly corresponds to the normal amount of noise in an experimental sample, and the other noise levels represent a multiplicative factor times this baseline level. Increased noise levels mimic sequencing at a lower coverage, where random sampling of the inputted DNA content has a more profound effect and leads to a wider spread of read counts for genomic bins with the same copy number state. Furthermore, the noise is dependent on the copy number state, with larger copy numbers associated with a larger noise level. Multiplicative noise is used to test the method in situations where the assumption of equal variance is violated.

An *in silico* dataset is generated by mixing simulated sequencing reads of tumor DNA and a healthy control. Raw sequencing data are created using CNV-Sim [26], a simulation software that can generate raw fastq files using a predetermined set of alterations. We simulate artificial copy number profiles of chromosome 1 in a manner similar to the method described above. We ensure that at least three alterations are introduced and that the segment lengths of the alterations are in $\{30, 31, \dotsc , 50\}$. We avoid introducing any alterations in genomic bins $\{230, 231, \dotsc , 300\}$ corresponding to the centromere (when using a 500 kb bin size), as approximately this region will generally be removed by traditional bioinformatic tools. Furthermore, we ensure that the introduced alterations do not start or end within 10 genomic bins of this region and 20 bins within the start and end of the chromosome. These copy number profiles are used as the input of CNV-Sim, and derived fastq-files are aligned to chromosome 1 of the human reference genome (version hg19) using bwa. To dilute the samples to the desired purity $P$, we mix simulated reads of tumor DNA (T) with reads of a healthy control (C), also provided by CNV-Sim. Let $N_{\textrm{target}}$ be the desired number of reads for a fixed coverage level. The mixtures are constructed by first subsampling T and C to contain T$_{\textrm{reads}}$ and C$_{\textrm{reads}}$, respectively, such that T$_{\textrm{reads}}$ + C$_{\textrm{reads}}$ = $N_{\textrm{target}}$, where


4 \begin{eqnarray*}& \textrm{T}_{\textrm{reads}} = \frac{N_{\textrm{target}} P}{\alpha}, \quad \textrm{C}_{\textrm{reads}} = N_{\textrm{target}} - \textrm{T}_{\textrm{reads}},\end{eqnarray*}

where $\alpha$ is the estimated purity of T, using liquidCNA [21]. Note that it is necessary to first estimate the purity of T, as tumor genomes are highly aneuploid often containing more DNA than a healthy genome, therefore, our definition of purity (% of tumor cells contributing DNA) is not the same as the % of reads that have tumor origin. In the estimation of $\alpha$, we use the fact that we have the ground truth available. In short, liquidCNA estimates purity by estimating the distance between peaks of the smoothed copy number distribution in a sample. The smoothing parameter of the density is chosen such that the number of peaks in the estimated density matches the true number of copy number states. Furthermore, the segment values in the estimation are taken as the median segment values, conditioning on the true partition. We discard the sample if the estimated purity is $<50\%$. The mixtures are then constructed by combining the reads using samtools merge. Note that the sample purity and coverage are approximate due to rounding errors in the subsampling step.

Finally, we construct a dataset of low-quality *in vitro* and *patient-derived* lpWGS data. First, we use lpWGS data derived from *in vitro* mixtures of two paired high-grade serious ovarian cancer (HGSOC) cell lines [27], as described in [21]. The samples are mixtures of a sensitive and resistant subclone and a healthy control of known proportions and have an average coverage of 1.3$\times$. One technical replicate of the samples containing only tumor DNA (100% purity) is used to establish a baseline for the evaluation, as these samples naturally offer the most precise measurement of the true copy number states of the used cell line. Multiple samples are available to be used as baseline, corresponding to different sensitive/resistant mixtures. To construct the low-quality lpWGS data, we downsample the data to contain $\sim$10 million reads using samtools -s, corresponding to an average coverage of 0.15$\times$. The samples are processed using QDNAseq in the same way as the baseline.

To evaluate our method on patient data, we analyze two cfDNA lpWGS samples from Patient 4 of [28]. For patient details, sample processing and sequencing information, see [28]. This patient with HGSOC had two cfDNA samples sequenced, one at diagnosis (cfDNA1) and one after relapse (cfDNA2), with an average coverage of 1.7$\times$ and 3.5$\times$. Purity estimates obtained from liquidCNA show that cfDNA1 has 6.5%, and cfDNA2 13% tumor content. We create downsampled samples taking 10%, 20%, or 50% of the original coverage using samtools -s, and process them together with the full sequencing data files using QDNAseq and BayesCNA.

### Processing of data

BCP is run using the bcp package, with 500 burnin iterations and $M = 2000$ MCMC iterations. We use the default $w_{0} = 0.2$ and set $p_{0} = 0.01$. This value of $p_{0}$ was selected in an initial tuning stage as it works in both low and medium purity, but we suggest a smaller $p_{0}$ if we know *a priori* that the sample is of higher coverage and/or purity, as this generally increases the precision, and a larger $p_{0}$ if the sample is noisy to increase the recall. Furthermore, we use the threshold $\varepsilon = 0.05$ for filtering the posterior changepoint probabilities and $\eta = 0.5$ in the merging step, for the synthetic and *in silico* datasets. For the lpWGS dataset, we use $\varepsilon = 0.05$ since diagnostic plots indicated that $\varepsilon = 0.1$ was too high.

We compare BayesCNA with the bioinformatical tools ichorCNA [18] and QDNAseq [16]. We run ichorCNA using the snakemake workflow available from within ichorCNA, using bin size 500 kb with the corresponding GC and mappability files, with a matched normal panel (PoN), no clonality estimation, and standard parameters otherwise. The PoN was constructed using 30 control samples of chromosome 1 generated by CNV-Sim, using the createPanelOfNormals.R script with the option chrNormalize=1 and GC, mappability and centromere files provided in ichorCNA. For QDNAseq, we use a binsize of 500 kb, genomic bins with mappability <75% and blacklisted regions are removed, using applyFilters. The functions estimateCorrection and correctBins are used for correction of GC and mappability biases. The normal sample is subsampled using samtools -s to contain the same number of reads as the mixture, and used to normalize the data using normalizeBins. For each comparison, we run BayesCNA on the data corrected and normalized by the corresponding tool to avoid variation originating from preprocessing and focus on differences in segmentation.

### Evaluation metrics

Let TP, FP, and FN be true positives, false positives, and false negatives, respectively. To evaluate the ability to detect CNAs, we compute the precision (positive predictive value; $\frac{\textrm{TP}}{\text{TP + FP}}$), recall (sensitivity; $\frac{\textrm{TP}}{\text{TP + FN}}$), and the F$_{1}$-score defined as


5 \begin{eqnarray*}& \textrm{F}_{1}\mathrm{-score} = 2 \cdot \frac{\textrm{Precision} \cdot \textrm{Recall}}{\text{Precision + Recall}}.\end{eqnarray*}

The F$_{1}$-score combines precision and recall into a single metric and penalizes models with imbalances between precision and recall. We define a TP as a predicted changepoint that falls within $\pm 2$ genomic bins within a true changepoint. No other measure than the location of the changepoint is considered, i.e. the direction and magnitude of the corresponding predicted CNA is not taken into account. If no changepoints are predicted (TP + FN = 0), we set the precision and the F$_{1}$-score to zero to reflect the method’s inability to detect changepoints (otherwise, the precision and the F$_{1}$-score is not defined). A predicted changepoint can only be assigned to a single true changepoint to prevent double counting of TPs, which would result in an inflated F$_{1}$-score.

For the lpWGS data, we instead study how much information is retrieved when reducing the coverage or purity of the samples. We use the normalized mutual information (NMI) metric:


6 \begin{eqnarray*}& \textrm{NMI}(X, Y) = \frac{I(X, Y)}{\sqrt{H(X) \cdot H(Y)}},\end{eqnarray*}

where $I(X, Y)$ is the mutual information of two segmentations $X$ (baseline) and $Y$ (reduced coverage sample), and $H(X)$ and $H(Y)$ correspond to the entropy of $X$ and $Y$, respectively. To obtain a fair measure regardless of the complexity of the variables, $I(X,Y)$ is normalized to lie in the interval $[0, 1]$. If NMI = 0 there is no correlation between the samples, while NMI = 1 indicates perfect alignment, i.e. perfect segment agreement. As with the other measures, the corresponding CNAs are not considered.

## Results

### Synthetic mixed populations

**Figure 2:**
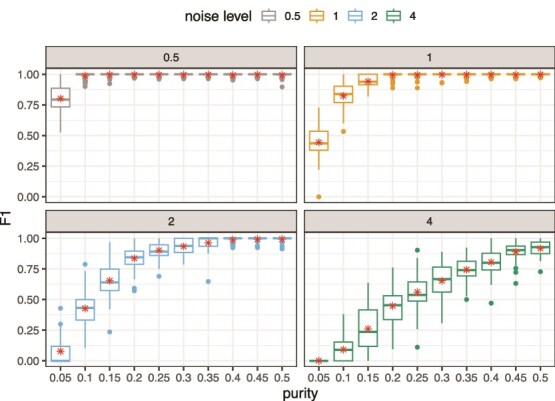
Predictive ability, measured as F$_{1}$-score, of BayesCNA in synthetic mixed populations, with added multiplicative Gaussian noise (increased noise levels represents decreased depth of coverage), with stars in the boxplot indicating the mean of the distribution.

We begin by evaluating the performance of BayesCNA on a dataset of synthetic copy number profiles with known noise distribution (Fig. 2). We generate this synthetic data with varying levels of noise and purity values. Increasing levels of noise, i.e. when observed binned read counts have a wider distribution, mimic decreased sequencing depth/coverage (Section Simulation of evaluation datasets). For each level of noise, we generate 50 samples per purity value $\{0.05, 0.1, \dotsc , 0.5\}$. We observe that the predictive ability decreases if either the purity or coverage is decreased, corresponding to an increase in the level of noise in the sample. We find a logarithmic like increase in the F$_{1}$-score as a function of the purity. However, higher noise levels cause the growth of the F$_{1}$-score to grow slower with increasing purity, because the signal is harder to distinguish from the noise in highly noisy scenarios. At noise level 0.5, the F$_{1}$-score is close to perfect, except for purity 0.05 and some outliers at higher purity levels. At noise level 1, we have an average F$_{1}$-score >0.75 with purities >5% while a purity of 20% is required at noise level 2. Finally, we find that the method breaks down for the highest noise level and that sufficiently high purities are required to retain the predictive ability.

### *In silico* mixtures

**Figure 3:**
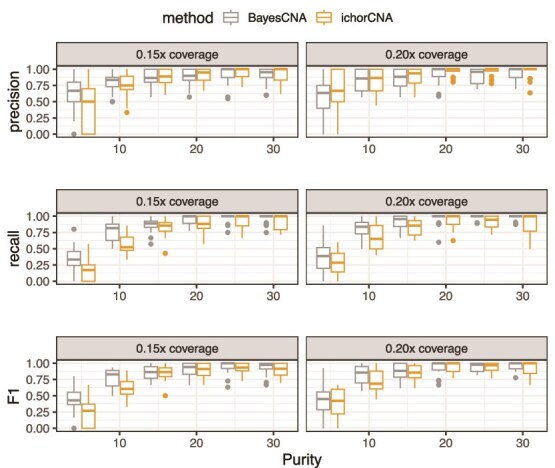
Comparison of changepoint detection ability between BayesCNA and ichorCNA, in terms of precision, recall, and the F$_{1}$-score.

**Figure 4:**
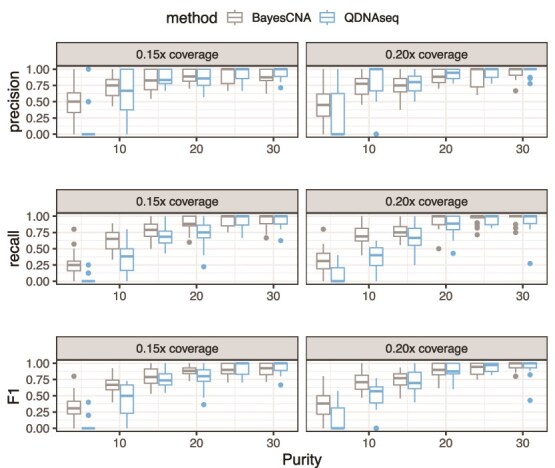
Comparison of changepoint detection ability between BayesCNA and QDNAseq, in terms of precision, recall, and the F$_{1}$-score.

**Figure 5:**
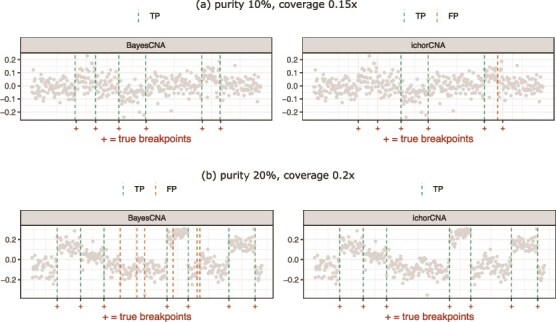
Illustrations of a case where BayesCNA performs (a) better, and (b) worse compared with state-of-the-art bioinformatical tool ichorCNA.

Next, we evaluate BayesCNA on a dataset derived from simulated raw sequencing files and compare its performance to ichorCNA and QDNAseq in terms of precision, recall, and the F$_{1}$-score (Figs 3 and 4). We use the software CNV-Sim to create tumor and normal fastq files and generate $20$ samples of each combination of purity $P \in \{0.05, 0.1, \dotsc , 0.3\}$ and coverage in $\{0.15, 0.2\}$ (Section Simulation of evaluation datasets). To ensure a fair comparison, we run BayesCNA on the preprocessed data produced by ichorCNA when comparing with ichorCNA, and on data preprocessed by QDNAseq when comparing with QDNAseq, as the normalization procedures differ between methods. In general, we find that the biggest advantage to using BayesCNA is in terms of recall, i.e. detecting more changepoints, especially for low-purity samples. The increase in recall is most evident when comparing the model with QDNAseq. Furthermore, the F$_{1}$-score for BayesCNA is at least on par with the state-of-the-art tools. Figure 5a presents a case in which BayesCNA performs better than ichorCNA. Here, we find that BayesCNA is able to detect all changepoints, while ichorCNA is only able to detect the most prominent segment. ichorCNA also detects the start of the rightmost segments but fails to accurately identify the end of the segment. We suggest that BayesCNA should be used in low-quality cases where a more sensitive method is required to identify genomic alterations. In Fig. 5b, we instead present a case where ichorCNA performs better than BayesCNA. In this case, BayesCNA introduced too many alterations (lower precision) while ichorCNA correctly classified all CNAs. Hence, our method might be more sensitive to “intra-segment” noise. However, noisy bins, or segments that are too short, can be removed in downstream analysis, while missed segmentation points cannot be recovered. We further note that the method was initially tuned to suit low-quality samples and that the selected parameters are suboptimal for high-purity cases.

### Ovarian cancer cell line and patient data

**Figure 6:**
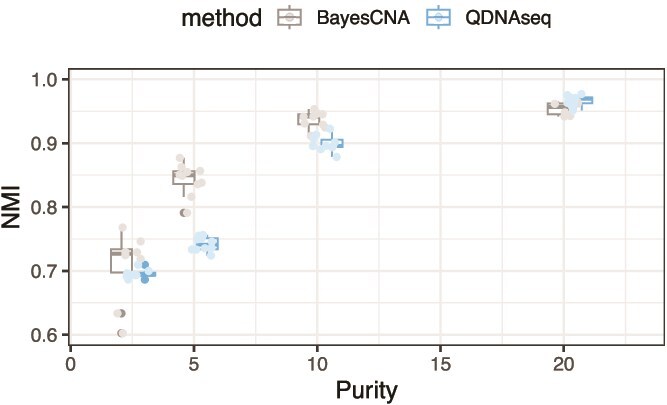
Comparison between QDNAseq and BayesCNA in terms of information retained in samples of experimentally decreased purity, measured as the NMI between high-quality baseline sample and downsampled (low coverage) samples.

Finally, we evaluate the performance and run-time of BayesCNA on experimental data obtained from ovarian cancer cell lines and a patient with ovarian cancer. First, we use *in vitro* cell line data to benchmark the performance of BayesCNA when sample purity is systematically decreased. As the true copy number states are unknown in this case, we instead rely on an established high-quality baseline sample, and measure the concordance, in terms of the NMI metric, between the segmentation of this baseline and low-coverage samples of the same DNA that have been experimentally diluted with non-tumor DNA (Fig. 6). As baselines for both BayesCNA and QDNAseq, we use the segmentation points detected in the low-noise and high-purity cell line sequencing data, as described in the Material and methods. We then evaluate how accurately the segmentation profiles are recovered from samples with lower tumor content and read count.

The mean run-time of BayesCNA is 3.6s (SD 0.29s) with the same settings as described in Section Processing of data, with the experiments performed on a Macbook Air M3, 16 GB RAM. We note that this time does not include the run-time for data-processing by QDNAseq. We observe that for purities close to $20\%$ (sample with $\sim 81\%$ purity not included), BayesCNA is on par with QDNAseq. In the most interesting region for this study, <10%, we find that the NMI scores for BayesCNA are significantly higher compared with the NMI scores of QDNAseq; except for purity values close to 2% where both methods perform poorly. However, we do not expect to extract any meaningful signal from samples with such low coverage and purity, since the inherent noise is on par with the signal, limiting the detection ability of lpWGS-based methods [17]. Furthermore, the method has not been tailored to such low-purity values (these purity values were not included in the tuning and evaluation process). At 5% purity, BayesCNA consistently shows NMI around 0.85, confirming that it can recover the majority of the information established in the high-quality baseline sample.

**Figure 7:**
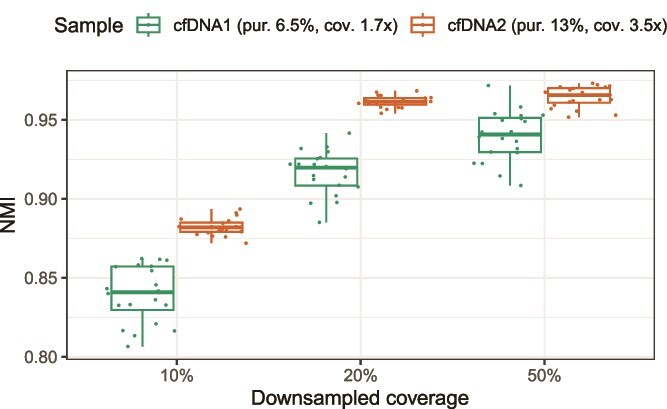
Performance of BayesCNA in terms of information retained in samples of decreased coverage, measured as the NMI between the original (full sequencing data) and downsampled samples.

Finally, we analyze two cfDNA samples from a patient with high-grade serous ovarian cancer, previously described as Patient 4 in [28]. The two samples were obtained at diagnosis and first relapse, and have coverage of 1.7$\times$ and 3.5$\times$ and purity of 6.5% and 13%, respectively. Since neither the true copy number profile nor high-quality baseline samples are available for these samples, we evaluate the output of BayesCNA when decreasing the coverage through downsampling using the full sequencing data as the basis of comparison. We find that BayesCNA can reliably recover information on almost all segment boundaries, even when the starting material is low quality and coverage is drastically decreased to 0.17$\times$ (Fig. 7).

In conclusion, we show that BayesCNA offers robust and consistent segmentation even when both the coverage and purity of samples is low (<0.2$\times$ and 3%–10%, respectively), showing it can be a valuable complementary/alternative for low-quality samples.

## Discussion

In this paper, we present BayesCNA, a computational method that segments and reconstructs copy number profiles from low-coverage sequencing measurements from liquid biopsies. Our method uses BCP detection to estimate posterior changepoint probabilities for each position in the genome to infer likely starts and ends of CNAs. A Bayesian approach is utilized because it naturally incorporates uncertainty, which is suitable for the noisy nature of liquid biopsy sequencing data. Using the detected partition of the genome, the signal is reconstructed by computing the segment medians conditionally on the partition. BayesCNA has been designed to increase recall (sensitivity) in low-coverage and low-purity samples where traditional methods struggle to detect any signal at all. BayesCNA works for any binned genomic data, regardless of the normalization procedure applied, and, therefore, can be easily incorporated into state-of-the-art pipelines to provide segmentation and copy number quantification.

We validate the method’s ability to detect changepoints using both synthetic and *in silico* liquid biopsy sequencing measurements. We find that BayesCNA can detect changepoints in sequencing data even if the underlying distributional assumption of the BCP model is not fulfilled. When comparing BayesCNA to the traditional bioinformatic tools QDNAseq and ichorCNA, we find that BayesCNA offers higher recall, especially when compared with QDNAseq. We further validate the model in terms of the F$_{1}$-score, where we find that the F$_{1}$-score is at least on par with traditional methods. Finally, we demonstrate in experimental samples that we retain more information—quantified in terms of NMI—when the noise level is increased due to low coverage, compared with QDNAseq.

Recent benchmarking work has shown that at purity of 10% or lower, lpWGS-based methods offer decreased performance in CNA detection [17]. Since we find that BayesCNA maintains a higher sensitivity in this regime, but can be prone to over-calling short segments, we suggest that it can be a complement to traditional methods to increase the ability to detect CNAs where these methods tend to break down. We believe its increased recall can be especially beneficial in liquid biopsy-based monitoring and discovery settings, where sensitivity is preferred over specificity, e.g. for detecting the emergence of novel cancer subpopulations. Besides liquid biopsies, our method could be deployed on other typical sources of low-quality samples, e.g. formalin embedded archival material, where short segment detection is anyways not advised due to formalin-induced artifacts [17]. Furthermore, BayesCNA can serve as segmentation/reconstruction step for downstream methods that utilize CNA profiles obtained from read depth information in their analyses. In particular, it might be best suited to provide segmentation for methods relying on large-scale (e.g. >6 Mbp) CNAs or summary statistics across entire genomes in their analyzes, where larger alterations are often of most interest, with short segments potentially filtered away. On the other hand, BayesCNA might be less suitable for analyses working with high resolution, such as deep-sequenced tissue biopsies, and where the accuracy of short segments plays a large role in prediction.

We suggest that model parameters should be selected based on prior beliefs about the data or based on visual inspection of diagnostic plots. For example, if we know that the sample is of high purity, we should prefer a smaller $p_{0}$ than used in this study and potentially filtering and merging of segments more aggressively. However, if the samples are of low quality, we should stick to less restrictive parameters to ensure that the signal is not lost in the post-processing. Furthermore, validation was only performed for alterations of length 30–50 genomic bins. We suggest that the parameters are tuned to suit the length distribution representing the sample at hand. We speculate that the method will generalize to longer segments, since this is generally a simpler problem, but shorter lengths require extra attention. Overall, as the runtime required for BayesCNA is negligible, we recommend to use it in parallel with multiple setting or other methods for obtaining segmented copy number profiles and thus benefit from its increased sensitivity for detecting alterations.

**Key Points**

- We develop a method, BayesCNA, for segmentation of (cancer) genomes and quantification of copy number alterations in liquid biopsy data based on Bayesian changepoint detection.
- We evaluate BayesCNA on synthetic and *in silico* data and show that it offers improved recall when tumor purity and coverage are low.
- We demonstrate on ovarian cancer cell line and patient data that BayesCNA can reliably recover copy number segment boundaries and profiles even in subpar samples.
- We recommend to use BayesCNA when the available data is of low quality (coverage and/or tumor content) and sensitive detection of all segment boundaries is the priority.

## Data Availability

Aligned sequencing data from ovarian cancer cell lines is available from the European Nucleotide Archive (ENA: PRJEB42332). Raw CN values for these samples are available from https://github.com/elakatos/liquidCNA_data. Sequencing data for the ovarian cancer patient data are available in the European Genome-Phenome Archive (EGA) at EGAS50000001142. The preprocessed copy number profiles are publicly available at https://data.mendeley.com/datasets/m93sk9n767/1. All code implementing BayesCNA and analyzing the results is available here: https://github.com/lottaer/BayesCNA
